# Fatalism in breast cancer and performing mammography on women with or without a family history of breast cancer

**DOI:** 10.1186/s12905-019-0810-6

**Published:** 2019-09-13

**Authors:** Maryam Molaei-Zardanjani, Mitra Savabi-Esfahani, Fariba Taleghani

**Affiliations:** 1 Faculty of Nursing and Midwifery, Isfahan University of Medical Sciences, Isfahan, Iran; 2 Department of Midwifery and Reproductive Health, Nursing and Midwifery Care Research Center, Faculty of Nursing and Midwifery, Isfahan University of Medical Sciences, Isfahan, Iran; 3 Faculty of Nursing &Midwifery, Nursing& Midwifery care research center, Isfahan University of Medical Sciences, Isfahan, Iran

**Keywords:** Breast cancer, Family history, Fatalism, Mammography

## Abstract

**Background:**

Breast cancer is the most prevalent cancer in women, and in those with a positive family history, it is important to perform mammography. One of the probable barriers in doing mammography is fatalism.

**Methods:**

This is a descriptive/cross-sectional study conducted on 400 women residing in Isfahan, Iran, randomly selected in 2017. Sampling was done randomly among the enrolled women in Health Integrity System. The data collection tool was a questionnaire regarding the demographic-fertility information and fatalism. The data analysis was done by SPSS software. A *P*-value < 0.05 was considered statistically significant.

**Results:**

The results showed that the mean rate of fatalism was 59.5 ± 23.2 in women with the experience of mammography, and 65.9±18.7 in women without the experience. Moreover, the mean rate of fatalism was 73.1±15.2 in subjects with a family history of breast cancer, and 59.3 ± 22.5 in those no family history related to this condition. Accordingly, fatalism was statistically significant associated (*P* < 0.001) with a family history of breast cancer and experience of mammography. There was no significant relationship between demographic information and fatalism (*P* > 0.05).

**Conclusion:**

The results indicated that fatalism in women with no experience of mammography was higher than in those with a positive history. Regarding the necessity of mammography in women with a family history of breast cancer, the required interventions seem to be essential to changing the viewpoints of women regarding the importance and effect of mammography as a screening method for breast cancer.

**Electronic supplementary material:**

The online version of this article (10.1186/s12905-019-0810-6) contains supplementary material, which is available to authorized users.

## Background

Breast cancer is the main cause of cancer-related mortality in women, hence a major health concern [1, 2]. The risk of women being affected by breast cancer is increasing, such that one in eight women contracts the disease [3].

Although the incidence of breast cancer is high in developed countries, the rate of mortality in less developed countries has been reported to be relatively higher, due to not diagnosing breast cancer at its earliest stages and lack of access to proper caring facilities [4]. Early diagnosis of breast cancer is an important process which increases the survival rate (SR) [5], and studies have shown that there will be a reduction in mortality rate in the next 15 years through screening [6].

The most important step in a timely diagnosis of the disease is screening. Breast cancer has the required criteria for screening and early diagnosis [7]. American Cancer Society suggests that for an early diagnosis of breast cancer, all women aged 40–44 years should undergo screening mammography on an annual basis [8]. Women with a positive family history of breast cancer are more likely to develop cancer [9]. In this regard, Braithwaite et al., (2018) reported a first-degree family history resulted in an absolute increase in 5-year risk of breast cancer [10].

Despite the effect of breast cancer screening on reducing mortality, some women still do not consent to mammography as a method of screening. This is due to the lack of awareness, concerns about the outcome of mammography, the unavailability of mammography from women’s point of view, the ostensible pain involved in the process, lack of knowledge on mammograms, negligence, lack of time, lack of understanding on the part of the spouse, and high costs [11–13].

One of the factors that may be negatively effective in the screening behavior is fatalism [14], considered as a socio-psychological factor in preventing cancer and fulfilling the screening behaviors [15]. Fatalism is the belief that conditions, such as disease or catastrophic events occur by a higher power (such as God), and cannot be avoided [14]. In fact, a doctrine that events are fixed in advance so that human beings are powerless to change them. (Webster’s Dictionary 2019) [16].

According to the results noted by Ghahramanian et al., (2016) 10.8% of women referred to health centers of Tabriz city in Iran, believed in fatalism. Moreover, the findings of some qualitative studies indicated that participating women mainly mentioned fatalism as a feeling of lack of control to prevent breast cancer [17, 18]. In this regard, Charkazi et al., (2013) showed that Iranian Turkmen women had high fatalism belief. They mentioned that fatalism is a significant belief in that society which could be considered as a barrier to breast cancer screening behaviors [15]. However, the results of Farmer et al., (2007) study showed that cancer fatalism was not as a direct correlate of mammography screening [19].

Although women without a family history may get breast cancer, but women with positive family history are at higher risk for getting breast cancer. To ensure that women, especially high-risk groups perform mammography for breast cancer screening, it is necessary to understand barriers that prevent women from having mammography.

Regarding the importance of mammography, especially in people with a positive family history of breast cancer, this study was done with the purpose of analyzing fatalism in breast cancer and mammography in women with or without a family history of breast cancer.

## Methods

The present is a descriptive/cross-sectional study with a one-stage plan. One of the 13 districts Isfahan, Iran, was selected on a random basis. Using Eq. 1, the sample size was calculated to be 400 people.

1 $$ n={\left(\frac{Z_{1-a/2}\times \delta }{d}\right)}^2=\frac{4\times {\delta}^2}{d^2} $$

Sampling was done randomly among the enrolled women in Health Integrity System (SIB). The inclusion criteria were women over 40 years of age, and minimum reading and writing literacy and more.

The data was collected using a self-administered structured questionnaire comprised of demographic-fertility questions and questions regarding fatalism in breast cancer (Additional file 1).

The demographic-fertility information in this questionnaire included age, number of children, and level of education, marital status, family history of breast cancer, and experience of mammography (*n* = 6).

The questions regarding fatalism were rated on a 5-item Likert scale (ranging from 1-strongly agree to 5-strongly disagree). Some examples of questions included “I believe if someone gets breast cancer, they will die soon”, “I believe if someone has a healthy diet, it cannot prevent breast cancer, they will get breast cancer”, “ I believe detection at early or advanced stages of breast cancer won’t make any difference, they will die from it”.

To determine the validity of fatalism questionnaire after studying the related books and papers, this questionnaire was given to 15 scholars and faculty members of the Nursing and Midwifery Faculty and the Faculty of Health in Isfahan University of Medical Sciences (IUMS). They reviewed the questionnaire for its content quality.

Test-retest method was used in the studied population to determine the reliability, with an interval of 2 weeks. Thus, the test was conducted at the beginning of the study and then 1 week later. The score of over 0.7 was considered reliable. The test-retest reliability was 0.8.

The study began after getting necessary permissions from Isfahan University of Medical Sciences-Iran, with ethical committee code 395782.

Participation in this study was also based on written informed consent. After the purpose of the study was explained to the women, the researchers received the letter of consent from the sample. Then the questionnaires were completed by the self-administered technique in a calm and private environment.

The obtained data in this study were analyzed by using the descriptive information and SPSS (Ver. 16) software.

## Results

The 400 women participated in this study. The majority of the women (55.7%) belonged to the 40–49 year age group, and 52.5% had four or more children. Most of the subjects (46.7%) had elementary education; 95% were married, and 15.5% had a positive family history of breast cancer (Table 1).

**Table 1:** Demographic and fertility information of the subjects in the study

Variable	No.	Percent
Age		
40–49	223	55.7
50–59	131	32.7
60–69	46	11.5
No. of children		
0–1	52	13
2-3	138	34.5
4 or more	210	52.5
Education		
Elementary	187	46.7
High school	183	45.7
University	30	7.5
Marital status		
Married	380	95
Single	11	2.8
Widow	9	2.2
Experience of mammography		
No	276	69
Yes	124	31
Family history of breast cancer		
No	338	84.5
Yes	62	15.5

The Pearson correlation coefficient showed no significant relation regarding the fatalism score between the women’s age (r = − 0.023, *P* = 0.65) and the number of children (r = 0.068, *P* = 0.17). Moreover, the results from the Spearman correlation indicated that the fatalism score had no significant relation with education in women (r = − 0.105, *P* = 0.13). The results further showed that there was no significant relation between fatalism score and marital status (r = − 0.21, *P* = 0.16) (Table 2).

**Table 2:** Correlation coefficient between demographic information and fatalism

Variables	R (Correlation Coefficient)	*P* Value
Age	−0.023	0.65
No. of children	0.068	0.17
Education	−0.105	0.13
Marital status	−0.21	0.16

There was no significant relationship between demographic information and fatalism (*P* > 0.05).

The mean rates for fatalism were 59.5 ± 23.2 in women with the experience of mammography, and 65.9 ± 18.7 in women with no experience of mammography. The statistical analysis showed that fatalism in women without the experience was significantly higher than those with the history of mammography (*P* < 0.001).

Furthermore, the mean score of fatalism in women with a family history of breast cancer was 73.1 ± 15.2, and that for subjects without the history was 59.3 ± 22.5. Fatalism had a statistically significant association with family history of breast cancer (*P* < 0.001) (Table 3).

**Table 3:** Mean and Standard Division (SD) of fatalism scores in different groups

Groups	Fatalism		t-test	*P*-value
	Mean	SD		
Family history of breast cancer				
Yes	73.1	15.2	21.51	< 0.001
No	59.3	22.5		
Experience of mammography				
Yes	59.5	23.2	7.24	< 0.001
No	65.9	18.7		

## Discussion

Our findings showed that the mean score of fatalism in women with no experience of mammography was higher. Thus, women believing in fatalism are less likely to undergo mammography. In this regard, Liang et al. [20] showed that the higher the belief in fatalism is, the lower the inclination towards screening for breast cancer will be, which is in line with the present study. Moreover numerous studies indicated that there is a relationship between health beliefs and behavior. They revealed screening rates were low among women with score highest on fatalism [21, 22].

This study also showed that fatalism is more common in women with a positive family history of breast cancer, which is in accordance with Tuzcu et al. [23]. They indicated that the belief in fatalism was higher in women with a family history of breast cancer than women without a family history. In this regard the results of the study by Opoku et al. [24] showed some participants believed breast cancer is an incurable disease and if someone gets breast cancer, they will die. The researchers mentioned that such beliefs are because many patients present for treatment at advanced stages and doctors are not able to treat them. In addition the findings of study Tracy et al. [25] showed that women who experienced a breast cancer survivor had more positive beliefs about mammography.

It seems that the lack of screening or detection at early stages of breast cancer in family members, and consequently, unsuccessful treatment of disease at advanced stages may lead to increased fatalism in women with a family history of breast cancer. Therefore, the results of present study may be due to inadequate understanding about breast cancer disease, as well the advantages of mammography as a screening technique in women.

Although the results of the study by Banning et al. [2] showed that fatalism was higher in people with lower education, in the current research, no significant relation was observed between the demographic factors (age, no. of children, education, and marital status); however, positive family history has been reported as one of the variables related to fatalism.

Cross-sectional basis and self-reporting by subjects were among the limitations of this research, hence the necessity of more extensive studies in this respect.

## Conclusions

Women with a family history of breast cancer, who have were more likely to believe in fatalism, may not consider mammography an effective screening method; thus, further interventions are recommended so as to change the viewpoints of women in this regard.

## Additional file

Additional file 1: Fatalism questionnaire (Persian and English versions). (DOCX 16 kb)

## Data Availability

The datasets generated during the current study are available from the corresponding author on reasonable request.

## Abbreviations

IUMS: Isfahan University of Medical Sciences
SIB: Health Integrity System
SR: Survival rate
