# Supplementary material for: Fatalism in breast cancer and performing mammography on women with or without a family history of breast cancer
Source: BMC Womens Health. 2019 Sep 13;19:116. doi: 10.1186/s12905-019-0810-6 (PMC6743202; doi:10.1186/s12905-019-0810-6)
Supplement: Supplementary file 1 — Fatalism questionnaire (Persian and English versions). (DOCX 16 kb) [file 12905_2019_810_MOESM1_ESM.docx]

| **Items** | **Strongly disagree** | **Disagree** | **Neither agree nor disagree** | **Agree** | **Strongly agree** |
| --- | --- | --- | --- | --- | --- |
| 1. I believe if someone has a healthy diet, it cannot prevent breast cancer, they will get breast cancer |  |  |  |  |  |
| 2. I believe if someone gets breast cancer it is the will of God. |  |  |  |  |  |
| 3. I believe if someone gets breast cancer, they will die soon. |  |  |  |  |  |
| 4. I believed people aren't willing to know about breast cancer because it is the manner they were meant to die. |  |  |  |  |  |
| 5. I believe if someone think to have cancer, they will get it. |  |  |  |  |  |
| 6. I believe chance of getting breast cancer doesn't depend on what people do. |  |  |  |  |  |
| 7. I believe detection at early or advanced stages of breast cancer won't make any difference, they will die from it. |  |  |  |  |  |
| 8. I believe if someone get cancer, they cannot do anything about it. |  |  |  |  |  |

|  | کاملا موافقم | موافقم | نظری ندارم | مخالفم | کاملا مخالفم |
| --- | --- | --- | --- | --- | --- |
| ۱- من معتقدم اگر کسی رژیم غذایی سالمی داشته باشد، این امرنمی تواند باعث جلوگیری  از ابتلا به سرطان پستان شود و آنها به سرطان پستان مبتلا خواهند شد. |  |  |  |  |  |
| ۲- من معتقدم اگر کسی به سرطان پستان مبتلا شود، خواست خدا می باشد. |  |  |  |  |  |
| ۳- من معتقدم اگر کسی به سرطان پستان مبتلا شود ، به زودی می میرد. |  |  |  |  |  |
| ۴- من معتقدم كه افراد نمی خواهند درباره سرطان پستان بدانند ،چون روش مرگ آنها مشخص می شود. |  |  |  |  |  |
| ۵- من معتقدم اگر کسی فکر کند که سرطان دارد، به آن مبتلا می شود. |  |  |  |  |  |
| ۶- من معتقدم که شانس ابتلا به سرطان پستان به کارهایی که افراد انجام می دهند بستگی ندارد. |  |  |  |  |  |
| ۷- من معتقدم که تشخیص سرطان پستان در مراحل اولیه یا پیشرفته تفاوتی نمی کند، چون فرد مبتلا به هرحال می میرد. |  |  |  |  |  |
| ۸- من معتقدم اگر کسی به سرطان مبتلا شود، نمی توانند برای او کاری انجام دهند. |  |  |  |  |  |
